# Photonic and Thermal Modelling of Microrings in Silicon, Diamond and GaN for Temperature Sensing

**DOI:** 10.3390/nano10050934

**Published:** 2020-05-12

**Authors:** Lukas Max Weituschat, Walter Dickmann, Joaquín Guimbao, Daniel Ramos, Stefanie Kroker, Pablo Aitor Postigo

**Affiliations:** 1 Instituto de Micro y Nanotecnología, IMN-CNM, CSIC (CEI UAM+CSIC) Isaac Newton, 8, Tres Cantos, E-28760 Madrid, Spain; j.guimbao@csic.es (J.G.); daniel.ramos@csic.es (D.R.); pabloaitor.postigo@imn.cnm.csic.es (P.A.P.); 2 Physikalisch-Technische Bundesanstalt, Bundesallee 100, D-38116 Braunschweig, Germany; stefanie.kroker@ptb.de; 3 Technische Universität Braunschweig, LENA Laboratory for Emerging Nanometrology, Universitätsplatz 2, D-38106 Braunschweig, Germany

**Keywords:** finite-element-simulation, optical ring resonator, diamond, silicon, gallium nitride, temperature sensor, thermal modelling, two-photon absorption, self-heating

## Abstract

Staying in control of delicate processes in the evermore emerging field of micro, nano and quantum-technologies requires suitable devices to measure temperature and temperature flows with high thermal and spatial resolution. In this work, we design optical microring resonators (ORRs) made of different materials (silicon, diamond and gallium nitride) and simulate their temperature behavior using several finite-element methods. We predict the resonance frequencies of the designed devices and their temperature-induced shift (16.8 pm K^−1^ for diamond, 68.2 pm K^−1^ for silicon and 30.4 pm K^−1^ for GaN). In addition, the influence of two-photon-absorption (TPA) and the associated self-heating on the accuracy of the temperature measurement is analysed. The results show that owing to the absence of intrinsic TPA-processes self-heating at resonance is less critical in diamond and GaN than in silicon, with the threshold intensity $I_{\text{th}}=\alpha /\beta$, α and β being the linear and quadratic absorption coefficients, respectively.

## 1. Introduction

Thermometry is one of the most elementary measurement techniques in physics, biology, chemistry and medicine. Since the invention of the well-known mercury-in-glass thermometer by Daniel G. Fahrenheit [1,2], thermometric devices were further developed to increase comparability, stability, reliability, and sensitivity [3,4]. Very precise instruments have been established in industry (e.g., the platinum resistance thermometer), albeit they are not easily miniaturizable and are susceptible to influences from the outside which can impair their calibration and temperature accuracy [5,6]. Considerable steps in that direction have been made by employing photonic structures like Optical Ring Resonators (ORRs) because of their high sensitivity to the ambient temperature [7,8]. The sensitivity of these sensing devices can be improved by combining high thermo-optic materials with the enhanced light-matter-interactions due to the high electromagnetic field in a resonator with high optical quality factor (Q-factor) [9]. Additionally, micro and nano-sized optical devices can be incorporated in biological applications [10], chemistry [11], microfluidic systems [12], lab-on-a-chip [13] and applications in outer space [14]. Therefore, accurate simulation and optimization of the performance, especially temperature sensitivity, is key prior to fabrication. Here we evaluate several methods for the simulation of ORR temperature sensors. We focus on models that require low computational resources and provide fast results. This is especially important for initial calculations making it easier and faster to study global parameters like dimensions or material properties. Simulation models in 2D and 3D were developed and compared to experimental data of a Si-based ORR, fabricated at NIST [8]. We use the finite-elements-method (FEM) [15] to calculate the relevant figures of merit: the free spectral range (FSR), the number of longitudinal modes (*m*) and the temperature sensitivity at the working wavelength λ=1550 nm.

In addition to the temperature sensitivity itself, self-heating effects are decisive for the feasibility of temperature measurements. Silicon is widely established as waveguiding material due to the high level of expertise in processing and its compatibility with the semiconductor industry [16,17]. Nevertheless, it shows some flaws that can be overcome by employing other materials. For instance, the recent work by H. Xu et al. [8] mentions the necessity to thoroughly understand the effect of material influence and TPA on the signal-to-noise ratio in temperature measurements with ultra-high sensitivity using silicon micro rings. The use of higher band gap materials (gallium nitride (GaN), diamond) would improve overall sensitivity and stability by minimizing propagation losses due to linear and non-linear absorption and the associated self-heating of the device. However, whereas the application of Si ORRs as electro-optical modulators is well studied [18], not many works can be found in literature employing microrings as efficient photonic thermal sensors. Furthermore, extending the study to materials such as GaN and diamond with excellent semiconducting and mechanical properties opens up opportunities to design composite opto-electronic and opto-mechanic devices, respectively [19,20,21,22].

## 2. Materials and Methods

In order to use the microring resonator as a temperature sensing device, the all-pass-filter configuration is employed [23]. It consists of a ring waveguide which is evanescently coupled to a bus waveguide [24]. The resonance condition in the ORR is:

(1) $$m\lambda_{\text{res}}=2\pi n_{\text{eff}}R_{0},$$

with *m* being the azimuthal mode number, $\lambda_{\text{res}}$ the resonance wavelength, $n_{\text{eff}}$ effective index, and $R_{0}$ the ring radius measured from the center of the ring to the center of the waveguide. The temperature shift of the resonance wavelength is given by the following equation, as derived by Xu [8]:

(2) $$\Delta \lambda_{m}=\left(\frac{\left(\frac{\delta n_{\text{eff}}}{\delta T}\right)+n_{\text{eff}}\left(\frac{\delta L}{\delta T}\right)\left(\frac{1}{L}\right)}{n_{\text{eff}}-\lambda_{m}\left(\frac{\delta n_{\text{eff}}}{\delta \lambda_{m}}\right)}\right)\left(\Delta T\times \lambda_{m}\right),$$

where $\Delta \lambda_{m}$ is the shift of the resonance on the wavelength-scale caused by an ambient temperature change ΔT, $\delta n_{\text{eff}}/\delta T$ is thermo-optic coefficient, L is the resonator length and δL/δT is the thermal expansion of the cavity. The denominator includes the group dispersion $N_{g}=n_{\text{eff}}-\lambda_{m}\left(\frac{\delta n_{\text{eff}}}{\delta \lambda_{m}}\right)$. To estimate the temperature sensitivity of the resonators, we use three different simulation approaches according to their computational effort required: axis-symmetric (AS), 2D and 3D. Modelling in 2D can provide accurate enough results to have an initial idea of what can be expected. Later refinement using 3D models can enhance the accuracy of 2D calculations.

**Figure 1:**
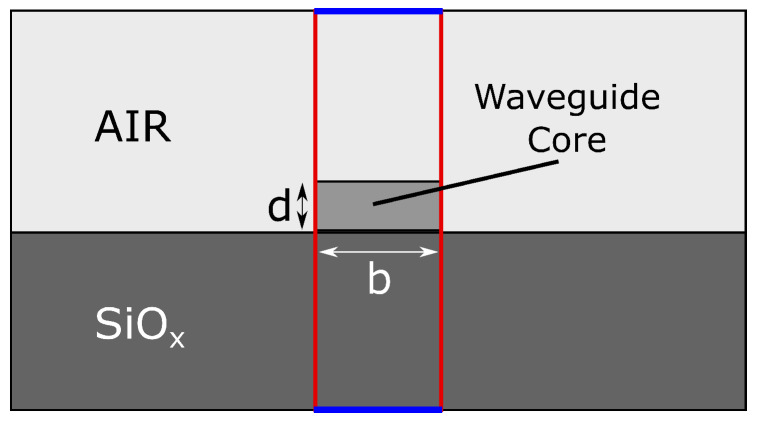
Layout of the waveguide cross-section. In case of the computation of the effective refractive index of the layer-system substrate-core-cladding, the Perfect-Magnetic-Conductor (PMC) boundary-condition is assigned to the side-boundaries of the layer-system (red), while keeping the Scattering-Boundary-Conditions (SBC) boundary-condition applied to the top and bottom borders (blue).

Figure 1 shows the cross-section of the waveguide (610 nm × 220 nm) made out of Si on a 2 μm thick SiOx substrate with air as cladding. We use the wavelength dispersion of Si from [25] and of SiOx from [26]. The dispersion of the air cladding is neglected due to its low value [27] and the strong confinement of the mode in the waveguide. To implement the thermal behaviour, the material’s thermo-optic coefficient is multiplied by the temperature difference ΔT and added to the wavelength-dependent core index. Prior simulations showed that the thermo-optic coefficients of the cladding (air) and the substrate (SiOx, 1.29 × $10^{-5}$ K${}^{-1}$ [28]) can be neglected for the same reasons as in the case of material dispersion. We find that the influence of the additionally grown 10 nm oxide layer (Ref. [8]) can be neglected as well since it only leads to an overall offset of the resonance peaks by Δλ≈2 nm and does not influence the temperature sensitivity. Regarding the thermal expansion coefficient (α(Si) = $3.57\times 10^{-6}$ K${}^{-1}$) we take the same approach as in Ref. [8] and neglect it since it is two orders of magnitude smaller than the thermo-optic coefficient of silicon ($2\times 10^{-4}$ K${}^{-1}$). The remaining ORR-parameter are $R_{0}\approx$ 11 μm and gap = 130 nm.

The following three sections describe the developed simulation models (axis-symmetric, 2D, 3D) and their codes in Comsol are included in the Supplementary Materials.

### 2.1. Axis-Symmetric Model

**Figure 2:**
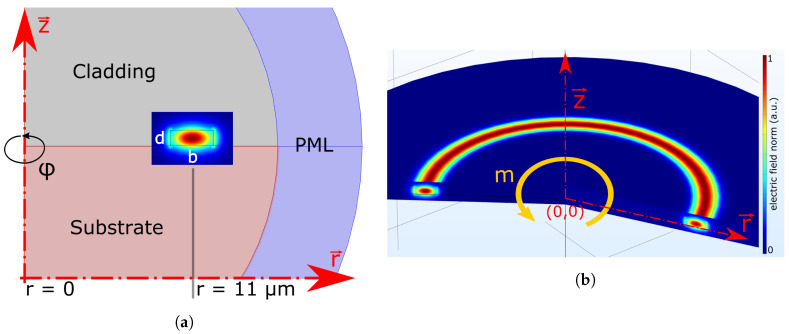
(**a**) Axis-symmetric model with axis of rotation at r = 0 and surrounded by a perfectly matched layer (PML)-domain. The fundamental TE-Mode is displayed in the ring waveguide b × d. (**b**) Resulting resonance of the axis-symmetric (AS)-model after computation, revolved around the z-axis using the azimuthal mode value *m* as input.

Axis-symmetric computations can be very fast and may provide enough accuracy during initial exploration of a number of parameters. The cross-section of the ring is modelled on top of the substrate using the mean radius $R_{0}$ and the waveguide dimensions. We use perfectly matched layer (PML) boundary conditions for the walls that enclose the whole design (inner wall radius = 1.5$R_{0}$, outer wall radius = 2$R_{0}$). This avoids light being reflected back into the system (Figure 2a). We define the azimuthal mode number *m* and the mesh-conditions (free triangular mesh with maximum element size of 55 nm within the waveguide core). The ring is formed by revolving the cross-section around the z-axis (Figure 2b). An eigenfrequency-study in the desired wavelength-regime is conducted to extract the FSR, resonance wavelength and their shift due to temperature.

### 2.2. 2D Model

**Figure 3:**
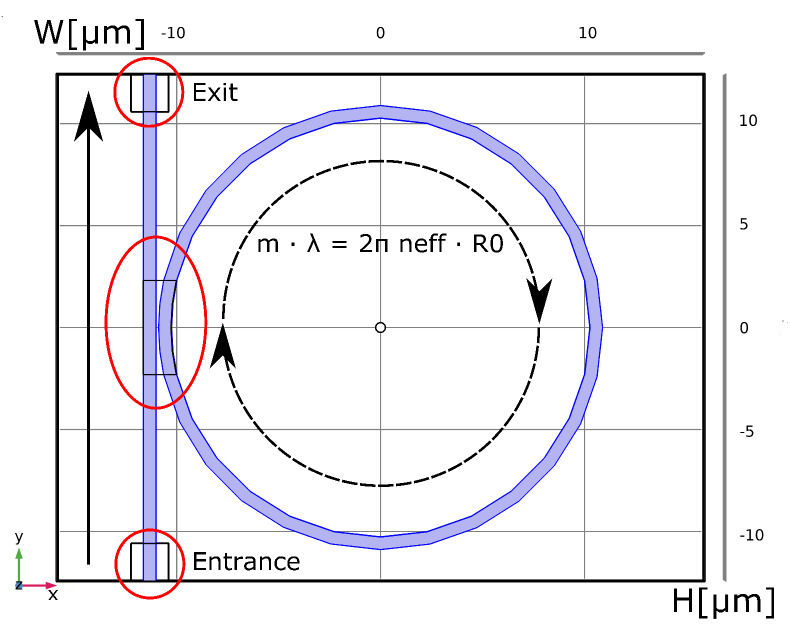
Layout of the ring resonator model with computation box of width W and height H [μm]. The entrance, exit and coupling region (red circles) have a refined mesh as they are critical for the performance of the resonator.

Simulations using a non-axis symmetric 2D approach can be performed for in-plane polarized light (TE) and employing an effective index. Figure 3 displays the layout of the structure. It consists of the ring and the bus waveguide which are enclosed by a computation box with dimensions W × H = $4R_{0}\times \left(2R_{0}+6b\right)$. We use Scattering-Boundary-Conditions (SBC) at the external boundaries except for the entrance and exit (rectangular port conditions). The maximum element size of the mesh is set to λ/5 to ensure that the mesh is fine enough to resolve the wave propagation, similar to the Nyquist-theorem [29]. The meshing is refined in the critical regions of entrance and exit ports and in the coupling section (red circles in Figure 3). To compute the effective index we use the waveguide cross-section shown in Figure 1 and assign Perfect-Magnetic-Conductor (PMC) boundary-conditions for the left and right boundaries (marked in red). A Frequency-Domain calculation predicts the transmission spectrum from which we extract the FSR and the resonance shift due to a temperature change. The 2D model has an advantage over the AS model if the electro-magnetic field distribution on and off resonance is of interest. Especially the coupling section and the ring radius have to be optimized to operate the ORR in ideal conditions (critical coupling, single mode, FSR [23]).

### 2.3. 3D Model

**Figure 4:**
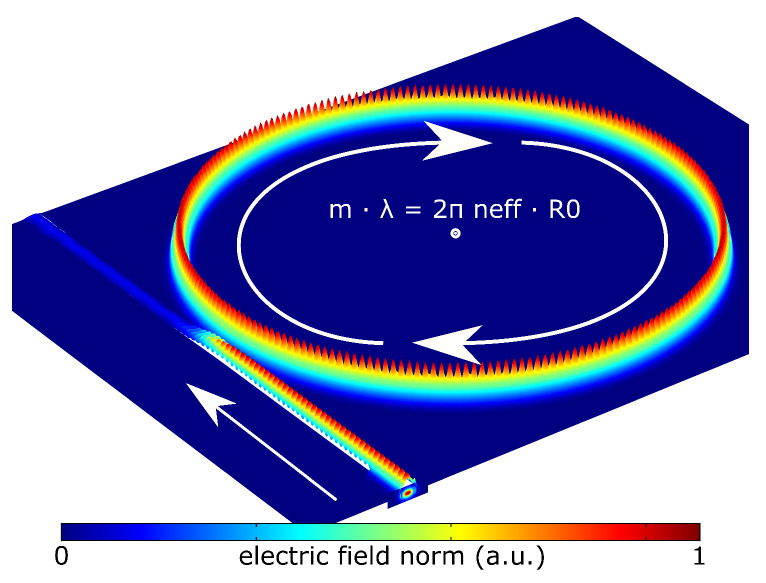
Electric field norm (Intensity) within the micro-ring resonator at resonance.

The layout in 3D follows the design in 2D (Figure 3) with an additional extrusion in the out-of-plane direction. We use SBC conditions for the computation box. The entrance and exit faces of the bus waveguide have the numeric-port condition. The maximum mesh size is set to λ/5 with refinements in the critical areas as in the 2D model. The numeric-port condition requires an additional Boundary-Mode-Analysis (BMA) step to find the propagating mode using the material refractive indices of the waveguide core, cladding and substrate. By doing so, we calculate the fundamental quasi-TE mode. Figure 4 shows the electric field norm (intensity) for the resonance wavelength using the 3D model as determined by the frequency-domain study. 3D modelling gives typically the most accurate results, however, we find that it takes roughly 80 times longer than in 2D and 35 times longer than in the AS-model.

## 3. Results and Discussion

### 3.1. Consistency Comparison of the Models

In order to compare simulation and experiment, we take the FSR, the azimuthal mode number *m* and the temperature sensitivity as figures of merit since they critically depend on geometric and material parameters (see Equation (1)). The mean radius of the Si-ORR is $R_{0}=10.485$μm, being $R_{0}\approx$ 11 μm in Ref. [8] and the calculated effective index of the waveguide is $n_{\text{eff}}=2.541$. With the 3D-model we obtain a resonance at 1549.8 nm (*m* = 108) that matches well with the calculated resonance using the AS-model (1548.24 nm). Table 1 compares the experimental values with the simulated results of the different models (AS, 3D, 2D).

**Figure 5:**
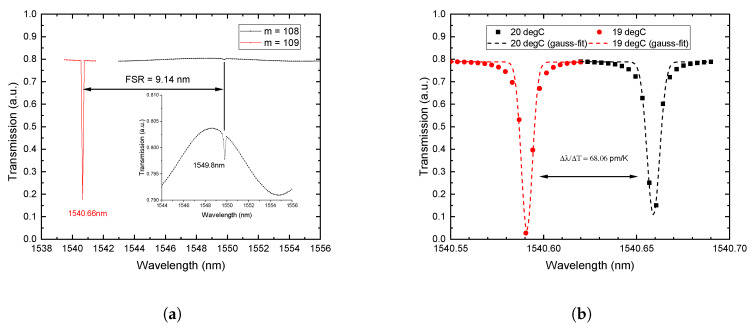
(**a**) Simulated transmission plot of the Si ring resonator (r_0_ = 10,485 nm, Waveguide (b × d) = 610 × 220 nm, gap = 130 nm) using the 3D-model. (**b**) Transmission of the Si-resonator resonance at 19 °C (red) and 20 °C (black).

We notice that in the AS model the mode number *m* is an input value to find the correct mode. However, in the 2D and 3D models *m* is an output that can be extracted from the spatial distribution of the electro-magnetic field. The FSR values calculated with the AS and 3D models are in good agreement with the experimental results. The small deviation can be attributed to the uncertainty of the ring radius and the refractive index in real experiments. The 3D-model provides the FSR closest to the experimental value (shown in Figure 5a). Calculated resonances for modes $m_{1}=109$ and $m_{2}=108$ using the 3D model are $\lambda_{1}$ = 1540.66 nm and $\lambda_{2}$ = 1549.8 nm, respectively. The inset in Figure 5a also reveals a Fabry-Pérot interference pattern in the transmission due to the simulated coupling waveguide (also present in the 2D model). Due to the absence of losses in the simulation the resonance peak becomes very narrow and the displayed extinction ratio strongly depends on the size of the wavelength steps taken during the simulation. Therefore, the wavelength resolution has to be chosen with care in order to resolve the resonances which further increases the computation time.

The FSR calculated from the 2D model deviates from the experimental value (FSR${}_{2D}$ = 12 nm, for m = 108 and m = 109). This is possibly due to 3 reasons: (1) the 2D model calculates with pure TE modes whereas the AS and 3D models give quasi-TE modes (full BMA), (2) the use of the effective index, and (3) the deviation of the effective index of a bent waveguide from the effective index of a straight waveguide. The last point plays a major role for the simulation of ring resonators. Since the whispering gallery mode is primarily guided by the outer edge of the ring, the field distribution deviates from a straight waveguide mode. Hiremath et al. [31] formulated an analytical approach to obtain the effective index of dielectric optical bent slab waveguides. Following their approach we calculated an effective index of $n_{\text{eff}}=3.2324$. This value was confirmed by a series of simulations sweeping the refractive index of the ring to find the index corresponding to the to the experimental FSR of ≈ 9.2 nm.

In an additional temperature parameter sweep the temperature behaviour of the modeled Si microrings is calculated. While the AS model calculates only the resonance frequencies, the 3D transmission results are shown exemplary in Figure 5b. Here the peak around 1540.66 nm (m=109) is displayed using the refractive index of Si at two different temperatures (ΔT = 1 K). Since the thermo-optic coefficient is positive the material index increases with temperature resulting in a resonance shift to higher wavelengths. The simulated data is fitted using gaussians (dashed lines) to determine the centre of the peak. The resonance shift per Kelvin (Δλ/ΔT) equals the temperature-sensitivity of the device. A slightly differing Si thermo-optic coefficient (1.6 × $10^{-4}$ K${}^{-1}$ [30]) than given (2 × $10^{-4}$ K${}^{-1}$ [8]) was used in the simulations. This is reflected in slightly lower values for the calculated temperature sensitivities (Δλ/ΔT≈ 68 pm K^−1^). In addition to that, we can determine a Q-factor of the Si-ORR from Figure 5b. With a FWHM ≈0.006 nm and the center wavelength $\lambda_{c}=1540.66$ nm, we estimate a Q-factor of Q $\approx 2.56\times 10^{5}$. Furthermore, we can calculate the dephasing time $\tau =\frac{2\hbar }{\Delta E}=\frac{1}{\pi \Delta f}=420$ps [32,33]. It has to be noted that the Q-factor and the dephasing time are estimates obtained from a simulation in which the losses (absorption, scattering due to sidewall roughness) are not fully taken into account and are therefore overestimated.

### 3.2. Temperature Sensitivity Comparison of Silicon, Diamond, Gallium Nitride

The axis-symmetric model was chosen to evaluate materials of choice as it provides adequate results for the sensitivity and short computation times. In this work we focus on materials with different thermo-optic coefficients at the wavelength of 1550 nm such as diamond (3 × $10^{-5}$ K${}^{-1}$ [34]) and GaN (5.2 × $10^{-5}$ K${}^{-1}$ [35,36]) and compare them to the silicon based ORR (1.6 × $10^{-4}$ K${}^{-1}$ [30]). In Section 3.3 we present an analysis in terms of the occurring self-heating effects in these materials. We design single-mode waveguides with similar propagation characteristics as in Ref. [8] for every material. Using effective refractive indices $n_{\text{e}}\text{ff}$ and the azimuthal mode number m=108 for a resonance at λ=1550 nm, the ring waveguide radii for every material are calculated using the resonance condition (Equation (1)). Since the AS-model is used the coupling gap distance is not needed as input value. The transmission spectra of the diamond and GaN-ORRs do not change substantially compared to Si-ORR (Figure 5a), therefore we do not show them here. Material properties and computed temperature sensitivities are summarized in Table 2.

**Figure 6:**
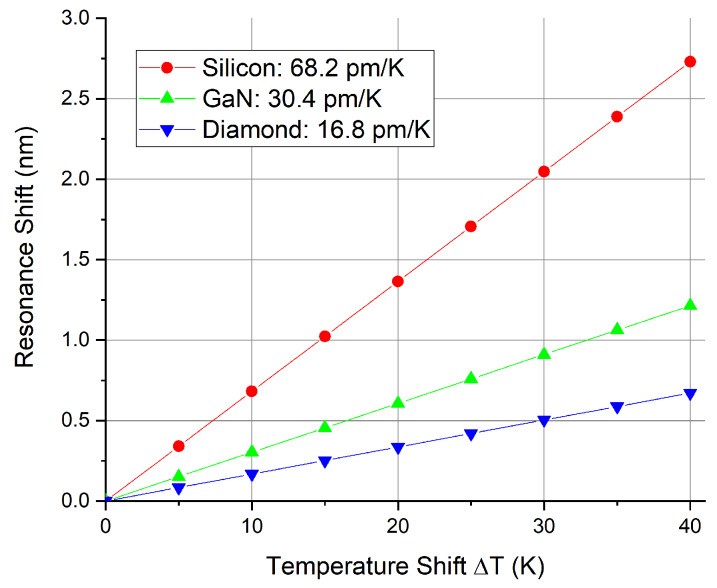
Computed sensitivities of ring resonators made of Si, gallium nitride (GaN) and diamond around 1550 nm using the axis-symmetric model. Reference temperature for the thermo-optic coefficient is room temperature.

Figure 6 shows the computed wavelength shifts of the resonance close to 1550 nm (m=108) depending on the ambient temperature for the investigated materials. Owing to its high thermo-optic coefficient, Si shows the highest temperature-sensitivity with an estimated shift of 68.2 pm K^−1^, followed by GaN with 30.4 pm K^−1^ and diamond with 16.8 pm K^−1^. As expected, the estimated temperature sensitivities follow the ratios between the thermo-optic coefficients. This indicates that the thermo-optic coefficient is the main contributor to the sensitivity. For the observed wavelength and temperature range (0–40 ${}^{\circ }C$) the thermo-optic coefficient can be considered linear. However, for larger temperature ranges it varies with temperature and decreases in general with lower temperatures (e.g., see [37] for the case of Si). In the this analysis the thermal expansion of the devices is neglected. For silicon this plays a minor role, however, the influence of thermal expansion on GaN and diamond is higher due to the smaller difference between the thermo-optic coefficient and the thermal expansion coefficient (Table 2). Since both coefficients lead to a resonance shift towards higher wavelengths with increasing temperature, the temperature sensitivity of GaN and diamond would be correspondingly higher. In addition to that, diamond has a high thermal conductivity, low thermal expansion and a wide bandgap [38,39], which predestines this material for opto-mechanical measurements.

The temperature sensitivity can be further increased if materials with even higher thermo-optic coefficients are employed. A promising material is Germanium (TO-coef.: 5.06 × $10^{-4}$ K${}^{-1}$ [25]) which would allow for estimated sensitivities of 175 pm K^−1^. However, due to its bandgap energy of only 0.6 eV it requires a longer operation wavelength (e.g., 2 μm). Calculation of sensitivities for other materials (e.g., organic and inorganic polymers which may be relevant for temperature biosensing [40]) is straightforward using the mentioned methods but here we restrict it to the three mentioned.

### 3.3. Self-Heating in Microrings

Absorption processes in optical ring resonators lead to local heating and thus influence the device performance. We developed an analytical model for the self-heating in optical ring resonators in order to estimate this effect for silicon, diamond and gallium nitride. For this purpose, we calculated the steady-state temperature rise due to optical absorption in the resonator and substrate. The steady-state temperature distribution is described by the steady-state heat equation [41]

(3) $$-\nabla^{2}T\left(x,y,z\right)=\frac{f(x,y,z)}{a},$$

where *T* is the temperature, *a* is the thermal diffusivity, *f* is the volumetric heat source and (x,y,z) are Cartesian coordinates. For a very large substrate volume, the solution of the steady-state heat equation in the substrate (referred to as *region 1*) is approximated as [42]

(4) $$T_{1}\left(x,y,z\right)=\int_{R^{n}}\frac{1}{a_{1}}\;T_{f}\left(x-x^{\prime },y-y^{\prime },z-z^{\prime }\right)\;f\left(x^{\prime },y^{\prime },z^{\prime }\right)\;d^{n}r^{\prime }+C$$

with the fundamental solution of the Laplace equation [42]

(5) $$T_{f}\left(x,y,z\right)=\left\{\begin{array}{c} -\frac{1}{2\pi }ln\left[\sqrt{y^{2}+z^{2}}\right]\\ \frac{1}{4\pi \sqrt{x^{2}+y^{2}+z^{2}}} \end{array}\right.\begin{array}{c} \mathrm{in}\;R^{2}\\ \mathrm{in}\;R^{3} \end{array}$$

and the dimension *n* of the problem. Please note that these fundamental solutions do not have the dimension of a temperature, as the actual temperature field $T_{1}\left(x,y,z\right)$ is calculated by a convolution on $T_{f}$ and *f* (see Equation (4)). The constant C is defined by the ambient temperature $T_{0}$.

The heat source is equal to the volumetric heat flux divided by the volumetric heat capacity [41] and has the dimension K/s. The heat source *f*_bus_ from the bus waveguide is approximated as a straight line source for the substrate temperature calculation. This approximation leads to

(6) $$f_{\text{bus}}\left(y,z\right)=bd\;\frac{\alpha I+\beta I^{2}}{\rho_{1}c_{m,1}}\;\delta \left[\left(y+\frac{D}{2}+b+g\right),z\right],$$

with the mean intensity *I* in the bus. The substrate mass density is $\rho_{1}$, the mass heat capacity is $c_{m,1}$, the linear attenuation coefficient of the bus material is α, the two-photon absorption coefficient is β, the waveguide width is *b*, the height is *d*, the ring radius is D/2, and the coupling gap is *g*. The term $\alpha I+\beta I^{2}$ represents the volumetric heat flux and $\rho_{1}c_{m,1}$ is the volumetric heat capacity. The corresponding temperature field $T_{1,\text{bus}}$ in the substrate due to the bus heat source is calculated by inserting Equations (5) and (6) into Equation (4) with n = 2. This leads to

(7) $$T_{1},\text{bus}\left(y,z\right)=-\frac{1}{2\pi }\;\frac{\alpha I+\beta I^{2}}{\lambda_{1}}\;\text{In}\left(\frac{{\left(y+\frac{D}{2}+b+g\right)}^{2}+z^{2}}{b^{2}}\right)+C$$

with the thermal conductivity $\lambda_{1}=a_{1}\rho_{1}c_{m,1}$ [43].

To compute the contribution of the ring resonator to the temperature rise, the respective heat source $f_{\text{res}}$ is approximated as a circular line source for the substrate temperature calculation. This approximation leads to

(8) $$f_{\text{res}}\left(x,y,z\right)=\pi Dbd\;\frac{\alpha I_{\text{res}}+\beta {I_{\text{res}}}^{2}}{\rho_{1}c_{m,1}}\delta \left[x^{2}+y^{2}={\left(\frac{D}{2}\right)}^{2},z\right]$$

where $I_{\text{res}}$ is the mean intensity in the resonator. The corresponding temperature field $T_{1,\text{res}}$ in the substrate due to the resonator heat source is calculated by inserting Equations (5) and (8) into Equation (4) with n=3. This leads to

(9) $$\begin{array}{cc} T_{1},\text{res}\left(x,y,z\right)=Dbd\;\frac{\alpha I_{\text{res}}+\beta {I_{\text{res}}}^{2}}{2\lambda_{1}} & \left[\frac{K\left[\frac{2\sqrt{x^{2}+y^{2}}D}{x^{2}+y^{2}+z^{2}+{\left(D/2\right)}^{2}+\sqrt{x^{2}+y^{2}}D}\right]}{\sqrt{x^{2}+y^{2}+z^{2}+{\left(D/2\right)}^{2}+\sqrt{x^{2}+y^{2}}D}}\right.\\ \; & +\left.\frac{K\left[-\frac{2\sqrt{x^{2}+y^{2}}D}{x^{2}+y^{2}+z^{2}+{\left(D/2\right)}^{2}-\sqrt{x^{2}+y^{2}}D}\right]}{\sqrt{x^{2}+y^{2}+z^{2}+{\left(D/2\right)}^{2}-\sqrt{x^{2}+y^{2}}D}}\right], \end{array}$$

where *K* is the complete elliptic integral of the first kind. The resulting temperature field is $T_{1}\left(x,y,z\right)=$ *T*_1,bus_(*y*,*z*) + *T*_1,res_(*x*,*y*,*z*). On resonance the influence of the bus waveguide on the temperature field is neglectable compared to the influence of the resonator due to $I\ll I_{\text{res}}$. Thus, the temperature rise in the substrate $\Delta T_{1}\left(x,y,z\right)=T_{1}\left(x,y,z\right)-T_{0}$ is approximately equal to *T*_1,res_(*x*,*y*,*z*).

In order to calculate the temperature distribution $T_{2}\left(x,y,z\right)$ in the resonator (referred to as *region 2*), we assume for the sake of simplicity a homogeneous intensity distribution $I_{\text{res}}\left(x,y,z\right)=I_{\text{res}}=const.$ in the resonator region 2. This leads to the volumetric heat source

(10) $$f\left(x,y,z\right)=\left\{\begin{array}{cc} \left(\alpha I+\beta I^{2}\right)/\left(\rho_{2}c_{m},2\right) & \mathrm{in}\;\mathrm{region}2\\ 0 & \text{elsewhere} \end{array}\right..$$

The Dirichlet boundary condition is determined by the heat transfer through the boundary δS between substrate and resonator that is described by

(11) $$\pi Dbd\left(\alpha I_{\text{res}}+\beta {I_{\text{res}}}^{2}\right)=h_{12}\pi Db\left(T_{2}\left(x,y,z\right)-T_{1}\left(x,y,z\right)\right){|}_{(x,y,z)\in \delta S}$$

with the thermal contact conductance h${}_{12}$ [44]. The left side of Equation (11) is equal to the totally absorbed power in the resonator. In thermal equilibrium, this power is in very good approximation completely transferred through the boundary δS into the substrate, as the thermal contact conductance between resonator and air is very low compared to the interface to the substrate [45]. The area of δS is equal to πDb and the resulting temperature difference is $\left(T_{2}\left(x,y,z\right)-T_{1}\left(x,y,z\right)\right){|}_{(x,y,z)\in \delta S}$. This leads to the boundary condition

(12) $$T_{2}\left(x,y,z\right){{|}_{(x,y,z)\in \delta S}=T_{1}\left(x,y,z\right)|}_{(x,y,z)\in \delta S}+\frac{d\left(\alpha I_{\text{res}}+\beta {I_{\text{res}}}^{2}\right)}{h_{12}}.$$

As the width of the resonator is very small compared to the resonator diameter (b≪D), the boundary conditions (12) can be linearized in radial direction. The solution of the resulting Dirichlet problem for the heat Equation (3) with constant source term (10) is approximately

(13) $$\begin{array}{c} T_{2}\left(x,y,z\right)=\frac{\alpha I_{\text{res}}+\beta {I_{\text{res}}}^{2}}{\lambda_{1}}\left(dz-\frac{1}{2}z^{2}\right)\;\\ +\frac{1}{2}\left[T_{2}\left(\sqrt{x^{2}+y^{2}}=\frac{D}{2}-\frac{b}{2},z=0\right)+T_{2}\left(\sqrt{x^{2}+y^{2}}=\frac{D}{2}+\frac{b}{2},z=0\right)\right]\;\\ +\frac{\sqrt{x^{2}+y^{2}}-D/2}{b}\left[T_{2}\left(\sqrt{x^{2}+y^{2}}=\frac{D}{2}+\frac{b}{2},z=0\right)-T_{2}\left(\sqrt{x^{2}+y^{2}}=\frac{D}{2}-\frac{b}{2},z=0\right)\right]. \end{array}$$

We briefly want to discuss Equation (13) and its consequences. The temperature field $T_{2}\left(x,y,z\right)$ is described by three terms: a z-dependent, a constant and a radial dependent term. The z-dependent term together with the constant term is the exact solution of the heat Equation (3) with constant Dirichlet condition on δS. The actual linearized Dirichlet condition is considered by the radial dependent term. The strongest temperature gradient in the resonator occurs in z-direction, as the total absorbed heat is transferred through δS at z=0. The heat transfer through the surface between resonator and air at z=d is neglected, which leads to $\delta_{z}T_{2}\left(x,y,z\right)=0$ at this surface. The much smaller gradient in radial direction is enforced by the linearized Dirichlet boundary conditions. Inserting $T_{2}\left(x,y,z\right)$ in the heat Equation (3) leads to a small deviation of the Laplacian of $T_{2}\left(x,y,z\right)$ from the heat source (10) due to the linear radial dependency. However, this deviation is at least three orders of magnitude smaller than the heat source itself for all considered materials: $-a_{2}\nabla^{2}T_{2}\left(x,y,z\right)/f\left(x,y,z\right)=1+\epsilon ,\;\epsilon <10^{-3}.$ Thus, the calculated resonator temperature field $T_{2}\left(x,y,z\right)$ is a good analytical approximation.

The calculation of the temperature field in a disc resonator can be performed in an analogous manner, but is not included here. It should be mentioned that these are the steady-state solutions that will be reached faster by disc resonators due to the improved thermal contact to the substrate.

We now discuss the self-heating effect for silicon, diamond and gallium nitride. Before applying the quantitative results to the three resonator materials, we briefly want to compare their absorption behaviour qualitatively. Silicon shows significant two-photon-absorption (TPA) [46,47], in the present wavelength regime [48] due to the extreme intensities within the ring waveguide at resonance [23]. This leads to an increased self-heating of the device for high input powers which consequently compromises the temperature measurement. Diamond and GaN are superior in this regard due to their wide band gaps, as their non-linear absorption would start to affect the device only at much lower wavelengths [49,50].

**Figure 7:**
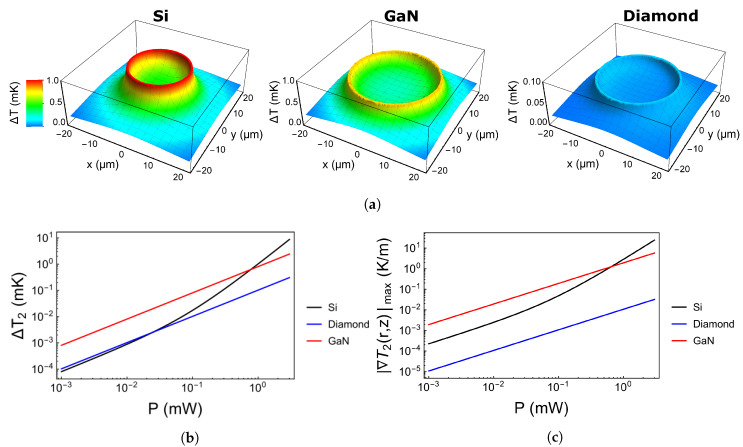
(**a**) Calculated temperature field in the Si, GaN and diamond resonators with a power of *P* = 1 mW. (**b**) Calculated mean temperature increase in the ring resonators as a function of the circulating power for Si, diamond and GaN. (**c**) Calculated maximum gradient of the temperature field in the ring resonators as a function of the circulating power for Si, diamond and GaN.

For each resonator material, we compute the temperature rise $\Delta T_{2}\left(x,y,z\right)=T_{2}\left(x,y,z\right)-T_{0}$ in the resonator and $\Delta T_{1}\left(x,y,z\right)=T_{1}$,_res_(*x*,*y*,*z*) in the substrate by applying Equations (9) and (13). The geometrical parameters of the resonator structures are listed in Table 2. The thermal conductivity of the substrate (fused silica) is λ^1^ = 1.3 W m^−1^K^−1^ [51] and the resonator material conductivities are λ_2,si_ = 130 W m^−1^K^−1^ [52], λ_2,c_ = 2200 W m^−1^K^−1^[52], and λ_2,GaN_ = 200 W m^−1^K^−1^ [53]. The thermal contact conductance across nanoscale silicon and fused silica interfaces is calculated and measured as h_12,si_ ≈ 10^9^ $Wm^{-2}$$K^{-1}$ [54]. For both other interfaces (diamond and GaN on fused silica) to the best of our knowledge no specific measurement or theory values for the thermal contact conductance are available. However, it is documented that for many other chemically bound interfaces the thermal contact conductance is in the range $\left[10^{8}\cdots 10^{9}\right]$$Wm^{-2}$$K^{-1}$ [55]. Thus we assume h_12,GaN_ ≈ 10^8^ $Wm^{-2}$$K^{-1}$ as the worst case scenario. The thermal contact conductance between diamond and other solids is documented to be significantly smaller compared to other interfaces [55]. We apply the lowest documented thermal contact conductance between diamond and another solid here (h_12,C_ ≈ 10^6^ $Wm^{-2}$$K^{-1}$) [56] to attain an upper limit for the self-heating effect. The two-photon absorption coefficient is close to zero for diamond and GaN at 1550 nm and $\beta_{\mathrm{Si}}=8\times 10^{-12}$$mW^{-1}$ for Si [48]. The linear attenuation coefficient strongly depends on the purity and crystal structure of the resonator material. Reasonable values for high quality resonators are α_si_ = 0.01 m^−1^ [57], α_C_ = 0.01 m^−1^ [58] and α_GaN_ = 0.01 m^−1^ [59]. The spatial dependent temperature rise ΔT(x,y,z) is shown in Figure 7a for Si, GaN and diamond with a power P=1mW within the resonator. The maximum temperature rise along the resonator ring is about 1 K. The mean temperature rise ΔT${}_{2}=\langle \Delta T_{2}\left(x,y,z\right)\rangle$ in the ring resonators as a function of the power *P* is depicted in Figure 7b for Si, diamond and GaN. The non-linear behaviour of Si due to two-photon absorption comes into play at the threshold intensity $I_{\text{th}}=\alpha_{\text{Si}}/\beta_{\text{Si}}$ at which linear and quadratic absorption show equal heating contributions. For low powers ($I_{\text{res}}<I_{\text{th}}$) Si is preferable due to a very low thermal contact resistance and a low linear attenuation coefficient. However, for high powers ($I_{\text{res}}>I_{\text{th}}$) diamond shows the lowest self-heating due to the wide band gap and thus negligible two-photon absorption.

Another potentially important value is the maximum gradient of the temperature field in the resonator $|\nabla T_{2}{\left(x,y,z\right)|}_{\max}$, as it corresponds to stress induced refractive index changes. This value is computed by applying Equation (13) and the results are depicted as a function of power in Figure 7c. Diamond is superior here due to its very high thermal conductivity. However, the resulting temperature difference within the resonator is only about $10^{-3}$ mK in Si for P=1 mW which is about three orders of magnitude smaller than the self-heating itself. Thus, temperature gradient effects are less critical for the device performance.

## 4. Conclusions

We investigate the temperature sensitivity of ORRs and the influence of self-heating due to two-photon absorption. We used three different finite-element-methods (AS, 2D, 3D) to calculate the FSR of the resonator and the shift in resonance due to the change in temperature. In terms of accuracy of the calculated figures of merit (FSR, resonance wavelength) the 3D and AS model are comparable while the 2D model has a greater deviation. The three models give similar temperature sensitivities. However, while the 3D-simulation can be quite demanding in terms of calculation power, the 2D-model introduces unnecessary complications due to the effective index. Due to its higher thermo-optic coefficient, Si exhibits the highest temperature sensitivity (68.2 pm K^−1^) and compared to diamond (16.8 pm K^−1^) GaN (30.4 pm K^−1^). However, the analysis in terms of self-heating shows that using diamond or GaN would improve the thermal behaviour of the resonator at high intensities. Compared to Si self-heating in diamond and GaN is less critical at intensities higher than $I_{\text{th}}=\alpha /\beta$ with the linear absorption and quadratic absorptions coefficients α and β, respectively. Furthermore, due to diamonds high thermal conductivity, low thermal expansion and a wide bandgap, it can endure the high optical power at resonance while also achieving high mechanical Q-factors [60] which also makes it a promising material for temperature sensing using opto-mechanics.

## Figures and Tables

**Table 1:** Comparison of the experimentally measured values [8] with the calculated ones using the different models (axis-symmetric (AS), 3D and 2D). (calc.)—calculated value, (i)—input value, (c)—counted value.

	NIST-ORR [8]	Si-ORR (AS)	Si-ORR (3D)	Si-ORR (2D)
	*Experimental*	*Simulated*	*Simulated*	*Simulated*
**Input**
n(λ)@1550 nm	n/a	3.4757 *	3.4757 *	3.4757 *
dn/dλ@1550 nm [μm^−1^]	n/a	−0.08 *	−0.08 *	−0.08 *
Thermo-optic Coef.@20 ${}^{\circ }C$ [1/K]	2 × $10^{-4}$	1.6 × $10^{-4\;†}$	1.6 × $10^{-4\;†}$	1.6 × $10^{-4\;†}$
**Output**
Azimuthal Mode Number *m*	108 (calc.)	108 (i)	108 (c)	108 (c)
FSR (around 1550 nm) [nm]	9.2	9.06	9.14	12
T-Sensitivity [pm/K]	77	68.2	68.06	67–70

References: * [25], † [30].

**Table 2:** Device parameters used for the simulation of Si, diamond and GaN ring resonators.

	Silicon	Diamond	Gallium Nitride
Refractive Index (λ = 1550 nm)	3.4757	2.4792	2.3169
Waveguide width [nm]	610	850	800
Waveguide height [nm]	220	300	350
Effective Refractive Index $n_{\text{e}}\text{ff}$	2.541	1.8562	1.7439
Ring Radius $r_{0}$ (m = 108@1550 nm) [nm]	10,485	14,353.27	15,277.56
Optical Path Length ($n_{\text{e}}\text{ff}\times 2\pi r_{0}$) [μm]	167.40	162.31	167.40
Thermo-optic Coef. @20 ${}^{\circ }C$ [1/K]	1.6 × $10^{-4}$	3 × $10^{-5}$	5.2 × $10^{-5}$
Thermal exp. Coef. @20 ${}^{\circ }C$ [1/K]	2.6 × $10^{-6}$	1 × $10^{-6}$	3.17 × $10^{-6}$
Temperature Sensitivity [pm/K]	68.2	16.8	30.4
FSR (near 1550 nm) [nm]	9.06	9.66	9.58

## Supplementary Materials

The following are available at https://www.mdpi.com/2079-4991/10/5/934/s1, The Comsol codes (Version: 5.4) used for simulations are included.

## Abbreviations

The following abbreviations are used in this manuscript:

TPA	Two-Photon Absorption
FEM	Finite Elements Method
PML	Perfectly Matched Layer
PMC	Perfect Magenetic Conductor
ORR	Optical Ring Resonator
FSR	Free Spectral Range
SBC	Scattering-Boundary Condition
BMA	Boundary Mode Analysis
